# Behind Base J: The Roles of JBP1 and JBP2 on Trypanosomatids

**DOI:** 10.3390/pathogens12030467

**Published:** 2023-03-16

**Authors:** Luiz Henrique de Castro Assis, Stephany Cacete de Paiva, Maria Isabel Nogueira Cano

**Affiliations:** Telomeres Laboratory, Department of Chemical and Biological Sciences, Biosciences Institute, São Paulo State University (UNESP), Botucatu 18618-689, SP, Brazil

**Keywords:** trypanosomatids, Base J, J-binding proteins

## Abstract

β-D-glucopyranosyloxymethiluracil (Base J) is a modified thymidine base found in kinetoplastids and some related organisms. Interestingly, Base J distribution into the genome can vary depending on the organism and its life stage. Base J is reported to be found mostly at telomeric repeats, on inactive variant surface glycoproteins (VSG’s) expression sites (e.g., *T. brucei*), in RNA polymerase II termination sites and sub-telomeric regions (e.g., *Leishmania*). This hypermodified nucleotide is synthesized in two steps with the participation of two distinct thymidine hydroxylases, J-binding protein 1 and 2 (JBP1 and JBP2, respectively) and a β-glucosyl transferase. A third J-binding protein, named JBP3, was recently identified as part of a multimeric complex. Although its structural similarities with JBP1, it seems not to be involved in J biosynthesis but to play roles in gene expression regulation in trypanosomatids. Over the years, with the characterization of JBP1 and JBP2 mutant lines, Base J functions have been targeted and shone a light on that matter, showing genus-specific features. This review aims to explore Base J’s reported participation as a regulator of RNA polymerase II transcription termination and to summarize the functional and structural characteristics and similarities of the remarkable JBP proteins in pathogenic trypanosomatids.

## 1. Base J in Model Trypanosomatids

Trypanosomatids comprise a diverse group of protozoan parasites of the class Kinetoplastids, among which are species of the *Trypanosoma* and the *Leishmania* genera, which possess dixenous development. Some of these trypanosomatids are pathogens of medical importance, causing diseases of a range of severity whose treatment and control methods are still precarious, urging the finding of new anti-parasitic drug targets [1]. Trypanosomatids, like other Kinetoplastida, present a single mitochondrion carrying circular and catenated DNA, the kinetoplast, that shows unique functionality and structure. Besides that, they also present some important features regarding genome organization and dynamics. Their genome is variable in size. The haploid genome can range from around 30 Mb in *Leishmania* sp. up to 55 Mb in *Trypanosoma cruzi*. Most of their genes are organized in large clusters, polycistronicaly transcribed and processed by *trans*-splicing [2,3,4].

Moreover, many aspects of transcriptional regulation are still under investigation, mainly due to the absence of canonical promoters in RNA polymerase II transcribed genes. Species such as *Trypanosoma brucei* and *T. cruzi* present a set of genes involved with virulence being transcribed at the subtelomeric position [5,6], transforming the chromosome ends into potential targets for anti-parasitic treatment. At the vicinity of the genes encoding virulent proteins (such as the VSG, variant surface glycoproteins, in *T. brucei*) and towards the end of the chromosome are the telomeres. They are arrangements of DNA and proteins crucial to cell cycle maintenance and important cellular processes such as cell aging, genome integrity, and nuclear arrangement [7,8]. Moreover, as in other eukaryotes, in trypanosomatids, subtelomeric, and telomeric sequences can be transcribed in long noncoding RNAs involved in telomere maintenance and, therefore, in genome stability and cell survival [9].

Bernards and collaborators first reported a modified nucleotide at the telomeres on trypanosomatids and closely related organisms. This nucleotide, later known as Base J, was thought to affect antigenic variation in the bloodstream form of *Trypanosoma brucei* [10]. Later, the nature of Base J’s structure was discovered by Gommers-Ampt and collaborators [11,12], opening the doors for new studies on its biosynthesis, genome distribution, biological functions, and their impact on genome dynamics.

Base J, or β-D-glucopyranosyloxymethiluracil, is a modified thymidine whose synthesis is divided into two steps. In the first step, two thymidine hydroxylases, either J-binding protein 1 (JBP1) or J-binding protein 2 (JBP2), at a specific location on the nuclear DNA, alter a thymidine into hydroxymethyldeoxyuridine (HOMedU). In the second step, the HOMedU is glycosylated by a β-glucosyl transferase and transformed into Base J [13]. 

In *T. brucei*, Base J was first observed at inactive VSG expression sites (ES) only in the bloodstream form of the parasite [14]. It replaces approximately 1% of the total genomic thymidine, mostly at telomeric sequences. Nevertheless, a small amount of Base J is also found between the polycistronic transcription units (PTU) at RNA polymerase II initiation and termination sites [15,16]. Differences in Base J distribution were reported in bloodstream forms of *T. brucei*. About 13% of thymidine was modified into Base J at purified telomeric repeats, whereas only 0.8% was in total DNA. In addition, in bloodstream forms, approximately 50% of Base J was estimated to be located at telomeric repeats [17]. Localization of this hypermodified nucleotide at silenced VSG’s ES led to the hypothesis that Base J would be involved in gene silencing [10]. Later, studies involving the depletion of Base J strengthened this premise [18]. However, a knockout (KO) line for JBP1, although it significantly decreased Base J levels, had not shown variation in the expression of silent VSG, putting aside the hypotheses that Base J would work as a gene silencer in *T. brucei* [19]. Yet, the presence of Base J at transcriptional termination sites in *T. brucei* and a histone variant, H3.V, which regulates transcription termination [20], indicated a possible regulatory effect of Base J over gene transcription. Schulz and collaborators showed that Base J and H3.V simultaneous ablation increases the antisense transcription of genes near transcriptional termination sites [21]. Another study by Reynolds and collaborators suggested that Base J and H3.V can independently act or synergistically to regulate transcription termination and expression of coding and noncoding RNAs in *T. brucei* [22]. Later, Kieft and collaborators identified a novel J-binding protein, named JBP3, as part of a multimeric complex together with protein phosphatase (PP1), a homolog of Wdr82 and a potential PP1 regulatory protein (PNUTS) [23]. This complex is similar to the mammalian PTW/PP1 complex related to transcription termination via PP1-mediated dephosphorylation of RNA polymerase II. Unlike the other JBPs, JBP3 does not play a role in Base J biosynthesis (an aspect explored later in the present text) but rather in transcriptional regulation. In *T. brucei*, PJW/PP1 complex regulates termination through JBP3–Base J interactions and dephosphorylation of proteins such as RNA polymerase II and termination factors via PP1 [23]. Disruption of JBP3 expression or other components of PJW/PP1 complex led to defects of RNA polymerase II termination at 3′end of PTUs. 

Similarly, Jensen and collaborators identified JBP3 in *L. tarentolae* extracts [24]. They demonstrated that JBP3 interacts with different protein complexes likely involved in chromatin modification/remodeling and, to a lesser degree, with an RNA-Polymerase-II-associated factor 1 complex (PAF1C). Therefore, *Leishmania* uses Base J and proteins involved in chromatin remodeling and transcriptional regulation to induce RNA polymerase II transcription termination. Furthermore, the authors showed that the ablation of JBP3 in *L. tarentolae* resulted in a substantial increase in transcriptional readthrough at the 3′ end of most PTUs. These results suggest that JBP3 might recruit one or more of these chromatin remodeling complexes to the J-containing regions at the end of PTUs, which halts the progression of RNA polymerase II transcriptional activity.

Although Base J is also primarily a modification found at the chromosome ends, in *T. cruzi*, it is found in all parasite life stages, with it being upregulated (~two fold) in the infective mammalian stage [14]. *T. cruzi,* like other trypanosomatids, shuttles between the mammalian (metacyclic trypomastigotes and amastigotes) and the insect/vector (epimastigotes) forms. Around a quarter of *T. cruzi*’s Base J content is localized at subtelomeric regions, which is enriched in life-stage-specific surface glycoprotein genes involved in the parasite’s virulence [25]. Moreover, Base J is also present within sequences flanking the PTUs in *T. cruzi*. The knocking out of the two enzymes that regulate Base J synthesis (JBP1 and JBP2) decreased Base J levels at transcription initiation sites, correlating with aincreased RNA polymerase II transcription and a genome-wide increase in gene expression. Therefore, in *T. cruzi*, Base J may act as an epigenetic factor regulating RNA polymerase II transcription [26].

Moreover, parasites belonging to the *Leishmania* genus present an interesting Base J occurrence and distribution profile. Prior investigations showed that 99% of all Base J that co-migrated with telomeric repeat-containing DNA in J-immunoblots and Southern blots of fragmented DNA were all Base J content localized at the telomeric regions. The meaning is that 99% of Base J in *Leishmania* should be located at parasite telomeres [27]. Whereas *T. brucei* can survive without Base J [16,28], *Leishmania tarentolae*, for example, seems to require Base J to survive since JBP1 knockout (KO) cells are not viable [29]. In contrast, JBP2 KO cells were initially viable, showing a progressive loss of a significant amount of Base J through several passages in culture [30]. Interestingly, when bromodeoxyuridine (BrdU), a thymidine competitor, was added to these cultures, the loss of Base J was enhanced, leading to cell death [30]. Therefore, one possible function of Base J at *Leishmania* telomeres is maintaining cell homeostasis, although no detectable telomeric phenotypes were described in parasites maintained in early passages [27]. Van Leeuwen and collaborators helped to uncover Base J’s function in *Leishmania*. They showed that out of the telomeres in *L. major* and *L. tarentolae*, the remaining Base J content had been confined at RNA polymerase II transcription termination sites [27]. Thus, it is plausible to assume that *L. tarentolae* JBP2 KO cells submitted to BrdU treatment died due to the overall Base J reduction over these genetic and experimental conditions. In addition, they also showed that the loss of Base J is accompanied by massive readthrough of normal RNA polymerase II transcriptional termination sites. Therefore, it is likely that Base J is required for proper transcription termination and that the absence of internal Base J is lethal for *L. tarentolae* due to the massive readthrough of transcriptional stops [31].

In contrast, in *L. major,* although the reduction of Base J by dimethyloxalylglycine (DMOG) also resulted in genome-wide transcriptional readthrough at convergent strand switch regions (cSSRs) and head–tail (HT) sites, it does not trigger cell death [32]. Thus, it seems that in *L. major*, Base J regulates RNA polymerase II transcriptional termination at the end of each PTU, preventing the generation of genome-wide antisense RNAs. 

## 2. Base J Biosynthesis

**Figure 1:**
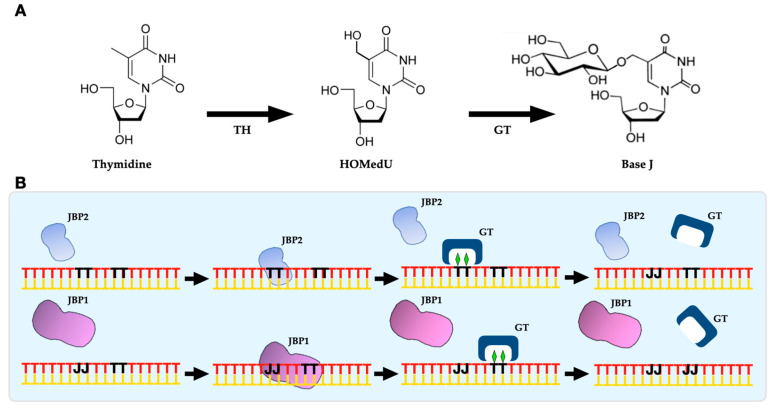
(**A**) Base J synthesis is divided into two distinct steps: (*i*) hydroxylation of a thymidine by a thymidine hydroxylase (TH), either JBP1 or JBP2, at a specific location on the DNA; (*ii*) the intermediate HOMedU is further glycosylated by a β-glucosyl transferase (GT) to become Base J. (**B**) Model of the synthesis and propagation of Base J in the trypanosomatids genome, in which JBP2 regulates the initial sites of Base J synthesis in bloodstream forms, with further propagation and maintenance of Base J by JBP1. In black, thymidine (T) and Base J (J). Figure adapted from Cliffe and collaborators, 2009 [28].

The presence of Base J only at specific sites replacing thymidine residues in the trypanosomatids genome was considered the first indication that Base J is a modified nucleotide and not a randomly incorporated mutated deoxynucleotide during DNA synthesis [13]. Therefore, studies about Base J functions rely on understanding its biosynthesis and the components that take part in it. As mentioned before, the modification of thymidine into Base J is thought to occur in two different steps (Figure 1). The first step involves the oxidation of thymine residues at its exocyclic methyl group. The oxidation is performed by a thymidine hydroxylase (TH) generating HOMedU (hydroxymethyldeoxyuridine), a Base J intermediate. Then, HOMedU is further glycosylated by a glucosyl transferase to form Base J (Figure 1A) [16,28]. The first step of Base J synthesis occurs via a J-binding protein, either JBP1 or JBP2, whose identification and characterization were carried out by Cross and collaborators and DiPaolo and collaborators, respectively [33,34]. 

JBP1 was first identified in nuclear extracts of *T. brucei* bloodstream forms, showing a specific binding activity to Base-J-containing duplex DNA. Similar results were also obtained with extracts of the non-pathogenic *Crithidia fasciculata* and *L. tarentolae*. In addition, recombinant JBP1 expressed in *Escherichia coli* also showed its specificity to bind Base J DNA content [33]. 

Sabatini and collaborators further explored JBP1 biochemical characteristics using J-DNA substrates and purified recombinant JBP1 protein [35]. They showed that JBP1 recognizes preferentially Base J when inserted in double-stranded DNA (dsDNA) but not in single-stranded DNA (ssDNA) or RNA–DNA hybrids. JBP1 also failed to interact with free Base J and cannot recognize and bind unmodified DNA or intermediates of Base J synthesis [35]. Later, Heidebrecht and collaborators went deeply into JBP1/J-DNA interactions. Based on the mutational analysis and hydrogen/deuterium-exchange mass-spectrometry data, they have shown that JBP1 recognizes J-DNA with 10,000-fold preference over normal DNA through a 160-residue domain, the J-DNA binding domain (J-DBD) [36]. Studies in *T. brucei* using JBP1 KO lines indicated that the disruption of JBP1 does not affect growth, gene expression, or the stability of some repetitive DNA sequences [19]. However, JBP1 KO lines present only about 5% of the wild-type levels of Base J in its nuclear DNA. Interestingly, excess Base J, randomly introduced into *T. brucei* DNA by offering the cells the Base J precursor HOMedU, is lost by simple dilution upon cell duplication [19], indicating that JBP1 does not protect Base J against its removal from the genome. These observations suggested that JBP1 contributes to Base J levels maintenance in the genome by using existent Base J to introduce additional Base J through its biosynthesis in regions of DNA that already contain the basal levels of this modified nucleotide (Figure 1B). 

Since JBP1 was shown to be a Base-J-specific DNA binding protein, the question of how the de novo synthesis of Base J occurs remained unanswered. DiPaolo and collaborators identified the homolog of JBP1, JBP2, which contains a domain related to the SWI2/SNF2 family of chromatin remodeling proteins, which is upregulated in *T. brucei* bloodstream form cells and interacts with nuclear chromatin. The expression of JBP2 in *T. brucei* procyclic cells leads to de novo Base J synthesis within telomeric regions. However, this activity is inhibited upon mutagenesis of conserved residues critical for SWI2/SNF2 function [34]. Consistent with these results, the knocking out of JBP2 in *T. brucei* bloodstream forms resulted in a decrease of five-fold in Base J levels and the inability of the parasites to stimulate Base J synthesis de novo in newly generated telomeric arrays [37].

Additionally, Yu and collaborators and Cliffe and collaborators showed that JBP1 and JBP2 belong to the family of Fe^2+^ and 2-oxoglutarate-dependent dioxygenases. In addition, the replacement of conserved residues putatively involved in Fe^2+^ and 2-oxoglutarate-binding inactivates the ability of JBP1 and JBP2 to contribute to Base J synthesis without affecting its ability to bind to J-DNA [15]. Thus, a model in which JBP2 regulates the initial sites of Base J synthesis in bloodstream forms was proposed with further propagation and maintenance of Base J by JBP1 [30,34,38] (Figure 1B). However, the nature of the glucosyl transferase that modulates the second step of Base J synthesis (JGT) remains debatable. 

A study by Bullard and collaborators shed light on this aspect of Base J synthesis [39]. Based on a computational screening, Iyer and collaborators [40] demonstrated that recombinant JGT utilizes uridine di-phosphoglucose to transfer glucose to HOMedU in the context of dsDNA. Deleting both alleles of JGT from the *T. brucei* genome generates a cell line lacking Base J. The *addback* of JGT into the JGT KO cell line restored Base J synthesis. Experiments using RNAi to promote the ablation of JGT mRNA led to the reduction of Base J and increased levels of HOMedU [39]. Similar results were observed in another study in parallel, using null mutants for JGT [41]. The analysis of the JGT function corroborates the two-step Base J synthesis model (Figure 1B), demonstrating that JGT is the only glucosyltransferase enzyme required for the second step of the pathway.

## 3. JBPs’ Structure

The structure, localization, and possible functions of Base J on the trypanosomatids genome have been explored since its discovery. However, many questions remain over the structure, activity, and identity of the three main enzymes that coordinate the thymidine turnover on Base J. Here we show information published in the literature on the JBP1, JBP2, and recently discovered JBP3 biochemical nature and structure that we could gather. As mentioned, JBP1 and JBP2 are members of the TET/JBP superfamily of 2-oxoglutarate-Fe+2-dependent dioxygenases that use Fe^2+^ and oxoglutarate as cofactors to hydroxylate pyrimidines [28,38,42]. In addition, these proteins have a characteristic double-stranded beta-helix fold domain (thymine dioxygenase domain) which is the catalytic core responsible for modifying bases in DNA [30,38,40]. Conversely, JBP3 has not been attributed to any specific protein family thus far; although, according to Kieft and collaborators [23], it shares with JBP1 a J-DNA-binding domain (J-DBD). 

The in silico structure of JBP1 and JBP2 can be found in protein databases such as UniProt (https://www.uniprot.org/ accessed on 3 March 2023) and AlphaFold (https://alphafold.com/ accessed on 3 March 2023). Unfortunately, no solved in vitro 3D structures are available for the full-length JBP1 or JBP2 proteins, but there are two entries at the Protein Data Bank (PDB: 8BBM and 2XSE) [36]. Both entries are for the *L. tarentolae* JBP1 J-DNA-binding domain (J-DBD) obtained through X-ray diffraction [43,44]. In contrast, the JBP3 protein is found after searching the UniProt code for putative proteins [23] since these databases have no annotation for JBP3 or its orthologs. On the other hand, it is possible to find 3D structure predictions for the entire JBP1 and JBP2 proteins in the AlphaFold protein database.

**Figure 2:**
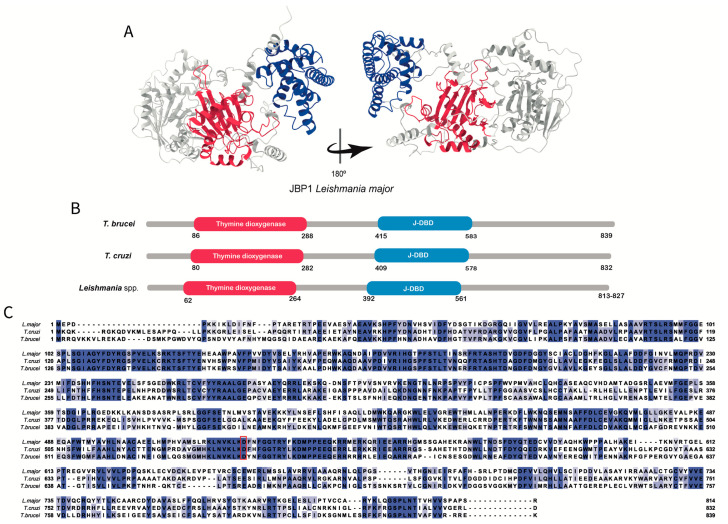
The JBP1′s structure: (**A**) Tridimensional *Leishmania major*’s JPB1 predicted structure obtained in AlphaFold database (Q4QHM7 (JBP1_LEIMA). The conserved domains thymine dioxygenase and J-DNA-binding domain (J-DBD) are shown in red and blue, respectively. (**B**) The domain’s positions in the amino acid sequence obtained in the UniProt database for *Trypanosoma brucei* (P86937), *Trypanosoma cruzi* (Q4DBW3), and *Leishmania major* (Q4QHM7). The conserved domains Thymine dioxygenase and J-DBD domain are shown in red and blue, respectively. (**C**) Multiple alignments were performed using the amino acid sequences obtained in the UniProt database for *Trypanosoma brucei* (P86937), *Trypanosoma cruzi* (Q4DBW3), and *Leishmania major* (Q4QHM7). The amino acids highlighted in different tones of blue, from light to dark blue, represent the percentage of identity (20–100%). The two histidine residues, the aspartic acid residue involved in Fe^2+^ binding, and the arginine residue important for binding 2-oxoglutarate are restricted by a black box [33]. Asp525, important for specificity, is highlighted in a red box.

**Figure 3:**
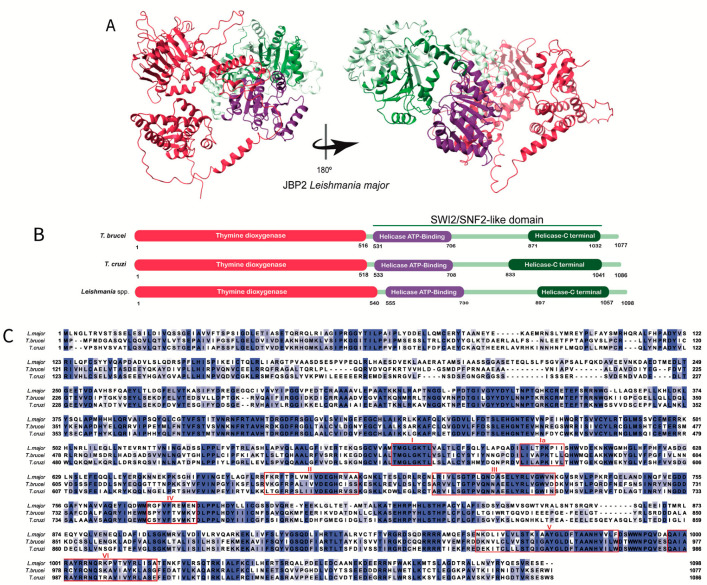
The JBP2′s structure: (**A**) Tridimensional *Leishmania major*’s JBP2 predicted structure obtained in the AlphaFold database (Q4QFY1 (JBP2_LEIMA). The conserved thymine dioxygenase domain (red) is depicted. The SWI/SNF2 domain is highlighted, and the subdomains (the helicase ATP-binding termianl and helicase C-terminal) are shown in purple and green, respectively. (**B**) The domain’s positions in the amino acid sequence obtained in the UniProt database for *Trypanosoma brucei* (Q57X81), *Trypanosoma cruzi* (Q4DCH3), and *Leishmania major* (Q4QFY1) are highlighted. The conserved thymine dioxygenase domain is shown in red. The SWI/SNF2 is depicted, and the subdomains (the helicase ATP- binding and helicase C-terminal) are shown in purple and green, respectively. (**C**) Multiple alignments were performed using the amino acid sequences obtained in the UniProt database for *Trypanosoma brucei* (Q57X81), *Trypanosoma cruzi* (Q4DCH3), and *Leishmania major* (Q4QFY1). The amino acids highlighted in different tones of blue, from light to dark blue, represent the percentage of identity (20–100%). The two histidines and the aspartic acid residues involved in Fe^2+^ binding and the arginine residue important for binding 2-oxoglutarate are restricted by a black box. The conserved motifs (I, Ia, II, III, IV, V, and VI) in the SWI/SNF2 domain are restricted by individual red boxes [34].

**Figure 4:**
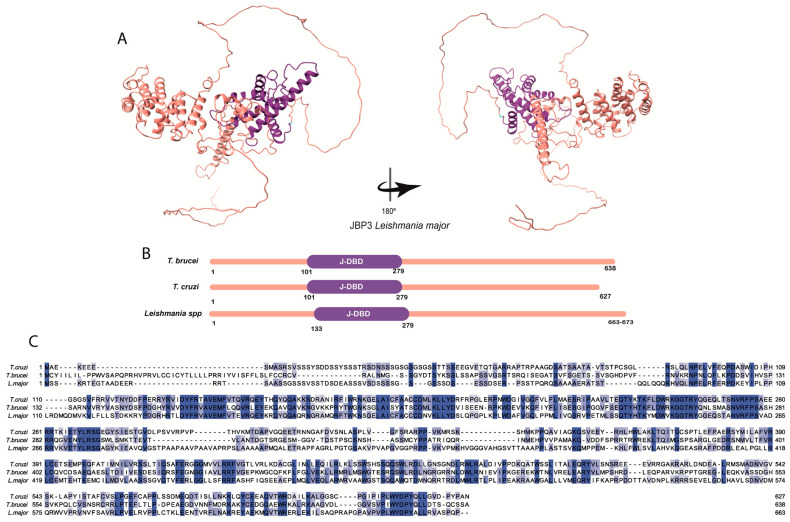
The JBP3′s structure: (**A**) tridimensional *Leishmania major*’s JBP3 predicted structure obtained in the AlphaFold database (Q4Q239). The conserved J-DBD domain (purple) is depicted. (**B**) The domain’s positions in the amino acid sequence obtained in the UniProt database for *L. major* (Q4Q239), *Trypanosoma brucei* (Q38BC1), and *Trypanosoma cruzi* (Q4CUX1) are highlighted. The conserved J-DBD domain is shown in purple. (**C**) Multiple alignments were performed using the amino acid sequences obtained in the UniProt database for *L. major* (Q4Q239), *Trypanosoma brucei* (Q38BC1), and *Trypanosoma cruzi* (Q4CUX1). The amino acids highlighted in different tones of blue, from light to dark blue, represent the percentage of identity (20–100%).Furthermore, the degree of conservation (identity versus similarity) between the three JBPs from the aligned species is low (about 32% for JBP1 and JBP2 and 19% for JBP3) (Table 1 and Figure 2C and Figure 3C). However, they all preserve the structural and functional domains involved in J synthesis and J-binding to DNA, indicating that the proteins retain their physicochemical properties, potentially allowing them to function similarly.

Representative structures for the three JBPs obtained through AlphaFold and UniProt are shown in Figure 2A, Figure 3A, and Figure 4A. It is important to note that these structures are predictions that need curation and that need to be confirmed through experimental testing. 

JBP1 and JBP2 are proteins with distinct functions that share 270 residues at their N-terminal region, comprising the thymine dioxygenase domain (Figure 2A,B and Figure 3A,B). According to Yu and collaborators, the thymine dioxygenase domain contains the all-beta core, a structural fold comprising eight beta strands conserved in all Fe^2+^/2-oxoglutarate-dependent dioxygenases [38]. This domain also shares residues with common dioxygenases: two histidines, one aspartic acid usually involved in Fe^2+^ binding, and an arginine important for binding 2-oxoglutarate (Figure 2C and Figure 3C). When these essential residues are mutated in JBP1, its ability to stimulate J synthesis is abolished, but it does not affect the ability to recognize and bind J-DNA [28,30,38]. On the other hand, the same mutation in JBP2 inhibits its capacity to stimulate de novo Base J synthesis [28]. The ability to recognize and bind J-DNA was demonstrated by Sabatini and collaborators [35]. Using a recombinant JBP1 protein, they showed its ability to specifically recognize Base J inserted in double-stranded DNA. However, as mentioned earlier, JBP1 cannot bind Base J in single-stranded DNA, RNA–DNA heteroduplexes, or as a free base. To bind J-DNA, JBP1 requires at least ten nucleotides with five flanking nucleotides on either side of Base J to form high-affinity complexes with J-DNA. Furthermore, JBP1 apparently can recognize the structure of the DNA helix, placing it in the category of structure-specific binding proteins [35]. However, unlike JBP2, JBP1 can recognize only and specifically Base J containing DNA [34,45]. This ability is due to the J-DBD in its C-terminal region (Figure 2A,B). A helix-turn-helix structure characterizes the J-DBD with an elongated turn between the helices [36]. The conserved Asp-525 residue in this domain (Figure 2C) is responsible for the specific recognition of J-DNA, and its mutation leads to the loss of this specificity [36,44,46]. In addition, Adamopoulos and collaborators showed that when JBP1 and J-DNA form a complex, a conformational change occurs, and the J-DBD domain becomes more defined and ordered [45]. 

Unlike JPB1, the C-terminal domain of JBP2 contains a SWI2/SNF2-like domain (Figure 3A). It is divided into two subdomains, a helicase ATP binding terminal and a helicase-C terminal (Figure 3B), responsible for recognizing and binding to certain chromosome regions, hydrolyzing ATP, and allowing the J-synthesis machinery to gain access to condensed chromatin by altering its architecture [30,34,37]. Dipaolo and collaborators showed that the SWI2/SNF2 of trypanosomatids JBP2 contains the canonical motifs (I, Ia, II, III, IV, V, and VI) and the specific residues implicated in ATP hydrolysis, conserved among the ATPase/DNA helicase family members (Figure 3C) [37]. Moreover, these domains are involved in chromatin remodeling since mutations in specific residues induce loss of the ATPase/chromatin remodeling function [28,34,37].

The structure of the JBP3 protein started to be characterized in *L. tarentolae* (sequence LtaP36.0380) by Kieft and collaborators using comparative modeling and alignment [23]. They described the J-DBD domain of JBP3 and demonstrated its high identity among different Kinetoplastida, concluding that this protein could be a J-binding protein due to its J-DBD domain. The domain composition of JBP3 can be seen in Figure 4B. Interestingly, when looking for the entries that Kieft and collaborators assumed to be JBP3 orthologs, we found an annotation describing an MYND putative domain in *L. major*. The MYND domain encompasses cysteine and histidine residues organized like fingers to form binding sites for Zinc or other metals [47]. Differently, the putative *Leishmania’s* JBP3 MYND domain is serine-rich, thus requiring further investigations.

We could not find information regarding the conservation and the comparative structural aspects of the JBPs over trypanosomatids species. Therefore, using public information on JBPs sequences, we performed amino acid sequence alignments among the three main human pathogenic species (Figure 2C, Figure 3C, and Figure 4C) using BLAST [48], Promals [49], and ENDspirit [50] (Appendix A). Analyzing the percentage of identity (invariant amino acids), we observed that the JBPs exhibit a high degree of conservation within the genus *Leishmania* (> 84% for JBP1, 89% for JBP2, and 83% for JBP3) but a comparatively lower degree of conservation among the *Trypanosoma* species (54% for JBP1, 56% for JBP2 and 40% for JBP3). This difference in conservation may indicate a higher selective pressure to preserve the primary structure of proteins in *Leishmania* than in *Trypanosoma*.

**Table 1:** Identity values of JBP1, JBP2, and JBP3 among different species of *Leishmania* and *Trypanosoma* genera.

% Identity JBP1						
	*L. major*	*L. tarantolae*	*L. braziliensis*	*L. infantu* *m*	*T. cruzi*	*T. brucei*
*L. major*	-	89.67	84.69	96.07	47.97	43.47
*L. tarentolae*		-	84.57	89.53	47.60	43.09
*L. braziliensis*			-	84.26	46.93	44.03
*L. infantum*				-	48.59	44.10
*T. cruzi*					-	54.03
*T. brucei*						-
Mean % identity: 32.11, mean % similarity: 70.20						
% Identity JBP2						
	*L. major*	*L. tarantolae*	*L. braziliensis*	*L. infantu* *m*	*T. cruzi*	*T. brucei*
*L. major*	-	94.35	90.07	97.09	47.58	43.51
*L. tarentolae*		-	89.34	94.63	47.58	43.45
*L. braziliensis*			-	90.44	47.77	43.64
*L. infantum*				-	47.77	43.90
*T. cruzi*					-	55.84
*T. brucei*						-
Mean % identity: 32.83, mean % similarity: 77.70						
% Identity JBP3						
	*L. major*	*L. tarantolae*	*L. braziliensis*	*L. infantum*	*T. cruzi*	*T. brucei*
*L. major*	-	87.92	87.23	96.07	40.73	31.47
*L. tarantolae*		-	83.61	87.76	40.91	31.41
*L. braziliensis*			-	85.44	40.21	33.16
*L. infantum*				-	40.62	31.48
*T. cruzi*					-	42.44
*T. brucei*						-
Mean % identity: 19.44, mean % similarity: 75.83						

## 4. Summary and Perspectives on Base J and JBPs

Base J is a unique and relevant modified nucleotide found in trypanosomatids. It is localized at subtelomeric and telomeric repeats and RNA polymerase II termination sites. The importance of Base J in different species of trypanosomatids has been accessed by investigating phenotypes of knockout lines for the enzymes producing Base J and by preventing Base J biosynthesis. For example, whereas *T. brucei* can survive without Base J, *Leishmania* spp. seems to require Base J to survive. Base J synthesis involves hydroxylation and further glycosylation of thymidine residues. The first step of Base J biosynthesis is catalyzed by two thymidine hydroxylases, JBP1 and JBP2, members of the Fe^2+^- and 2-oxoglutarate-dependent oxygenase family. While JBP1 is a J-DNA-binding protein (it presents a J-DNA-binding domain, J-DBD, in its C-terminal region) mediating the propagation/maintenance of Base J in the genome, JBP2 appears to be mainly responsible for de novo and site-specific Base J synthesis. The JBP2 SWI2/SNF2-like domain, in turn, recognizes and binds to certain chromosome regions, hydrolyzes ATP, and allows the J-synthesis machinery to gain access to DNA by chromatin remodeling.

Regarding their structure, JBP1 and JBP2 are considerably conserved, especially within the *Leishmania* genus. An additional J-binding protein was recently described in *T. brucei* and *L. tarentolae*, the JBP3. Although unrelated to Base J biosynthesis, JBP3 regulates gene expression in trypanosomatids by binding J-DNA with a multimeric protein complex involved with chromatin remodeling.

Much has been done to uncover Base J’s relevance in trypanosomatids gene regulation. Nevertheless, much needs to be done to answer important questions. Amongst them, we can mention (*i*) how specific thymine residues are targeted for J-modification. Would that be a random mechanism, or do unknown protein factors control it during development or environmental changes? (*ii*) Would the mechanism of J regulating transcription termination be genus- or species-specific? Is there a connection with parasite virulence/pathogenicity? (*iii*) Is the massive presence of Base J at *L. major* telomeres indeed involved with the transcriptional regulation of telomeric lncRNA such as TERRA? [51] (*iv*) What is the function of the antisense transcripts detected from regions upstream of transcription initiate sites? Are they only the result of bi-directional transcription activity? Which factors control their degradation since they are rapidly degraded and very hard to be detected? (*v*) If JBP complexes are similar in composition to TETs in higher eukaryotes, why do they differ in composition between *L. tarentolae* and *T. brucei*? Is that a rule depending on the genus and parasite species? Finally, how important are these findings concerning base J as an epigenetic factor?

The present review aimed not only to show the state of the art of our knowledge of Base J and J-proteins’ functions but to show that much has yet to be accomplished in the field. Base J is one of the few known epigenetic factors controlling gene expression in some trypanosomatids. Its absence in some *Leishmania* species suggests that JBPs may play extra roles in parasite biology. Therefore, those mentioned above and many other unanswered questions deserve more attention from the parasitology community.

## Data Availability

Not applicable.

## Appendix A

**Multiple sequence analysis:** Amino acid sequence alignments were performed using PROMALS [49] with default parameters in all cases. The sequences aligned were searched in UNIPROT and corresponded to JBP1 orthologs from *L. major* (Q4QHM7), *Trypanosoma brucei* (P86937), and *Trypanosoma cruzi* (Q4DBW3); JBP2 orthologs from *L. major* (Q4QFY1), *Trypanosoma brucei* (Q57X81), and *Trypanosoma cruzi* (Q4DCH3); and JBP3 orthologs from *L. major* (Q4Q239), *Trypanosoma brucei* (Q38BC1), and *Trypanosoma cruzi* (Q4CUX1).

**Identity and Similarity analysis:** To analyze the identity between the amino acid sequences, we obtained in UniProt the JBP1 sequences of *L. major* (Q4QHM7), *L. tarantolae* (Q9U6M1), *L. infantum* (A4HU70), *L. braziliensis* (A4H5X5), *Trypanosoma brucei* (P86937), and *Trypanosoma cruzi* (Q4DBW3); the JBP2 sequences of *L. major* (Q4QFY1), *L. tarantolae* (B6EU02), *L. infantum* (A4HVU6), *L. braziliensis* (A4H7G5), *Trypanosoma brucei* (Q57X81), and *Trypanosoma cruzi* (Q4DCH3); and JBP3 *L. major* (Q4Q239), *L. tarantolae* (LtaP36.0380), *L. infantum* (A4ICU8), *L. braziliensis* (A4HNP7), *Trypanosoma brucei* (Q38BC1), and *Trypanosoma cruzi* (Q4CUX1). We used BLAST to search each sequence and collect the % of identity. To obtain the % of identity and similarity, we performed multiple amino acid sequence alignments using PROMALS [37] with default parameters in Esprit (ESPript—https://espript.ibcp.fr) to collect the data.
